# The neutrophil-lymphocyte ratio predicts all-cause and cardiovascular mortality among United States adults with COPD: results from NHANES 1999–2018

**DOI:** 10.3389/fmed.2024.1443749

**Published:** 2024-09-25

**Authors:** Zhao Chen, Wenqiang Li, Yuanchun Tang, Peng Zhou, Qian He, Zhiping Deng

**Affiliations:** ^1^Department of Clinical Medicine, North Sichuan Medical College, Nanchong, China; ^2^Department of Respiratory and Critical Care Medicine, Zigong First People’s Hospital, Zigong, China; ^3^BGI College and Henan Institute of Medical and Pharmaceutical Sciences, Zhengzhou University, Zhengzhou, China; ^4^Department of Basic Medical Sciences, Changsha Medical University, Changsha, China; ^5^Department of Obstetrics and Gynecology, West China Second University Hospital, Sichuan University, Key Laboratory of Birth Defects and Related Diseases of Women and Children (Sichuan University), Ministry of Education, Chengdu, Sichuan, China

**Keywords:** chronic obstructive pulmonary disease, lymphocytes, neutrophils, mortality risk, NHANES

## Abstract

**Background:**

Neutrophil-to-lymphocyte ratio (NLR) is considered a biomarker of systemic inflammation and immune activation. However, its relationship with the risk of mortality in patients with chronic obstructive pulmonary disease (COPD) remains unclear. This study aimed to investigate the association between NLR and the risk of all-cause and cardiovascular mortality in patients with COPD.

**Methods:**

Data were collected from the National Health and Nutrition Examination Survey (NHANES) from January 1999 to December 2018. The calculation method of NLR involves dividing the neutrophil count by the lymphocyte count in the total blood cell count. The optimal NLR threshold associated with survival outcomes was determined using the maximally selected rank statistics method (MSRSM). The relationship between NLR and the risk of all-cause mortality and cardiovascular mortality in COPD was investigated using a weighted multivariable Cox regression model. Additionally, restricted cubic spline (RCS) was employed to discuss the potential relationship between NLR patients in different groups and the risk of mortality.

**Results:**

In this study, 716 adults with COPD were included using the maximally selected rank statistics method, among whom 208 had higher NLR (≥2.56) and 508 had lower NLR (<2.56). During a median follow-up of 111.5 months, 162 COPD patients died from all causes, and 49 patients died from cardiovascular diseases. After adjusting for demographic, socioeconomic status, and lifestyle factors, the risk of all-cause mortality (HR = 2.07, 95%CI: 1.46–2.94) and cardiovascular mortality (HR = 3.03, 95%CI: 1.63–5.65) in patients with higher NLR was increased by 2–3 times compared to those with lower NLR. Kaplan–Meier analysis revealed significantly lower survival rates in patients with higher NLR for all-cause mortality and cardiovascular mortality (*p* < 0.05). Restricted cubic spline analysis showed a linear correlation between NLR and the risk of all-cause mortality and cardiovascular mortality.

**Conclusion:**

NLR has a high value in independently predicting long-term all-cause and cardiovascular mortality risks in community-dwelling COPD patients. Therefore, NLR can serve as a cost-effective and widely available indicator for assessing the prognosis of COPD patients.

## 1 Introduction

Chronic obstructive pulmonary disease (COPD) affects approximately 4% of the global population, rising to 10% in those aged 40 and above (1). Currently, COPD stands as the third leading cause of death worldwide, with its global health burden increasing (2). Predictive models, such as the one by Boers et al. (3) suggest that by 2050, global COPD cases will approach 600 million. Most COPD patients have at least one clinically relevant chronic disease. While respiratory failure claims the lives of the most severely obstructed patients, the majority of COPD-related deaths stem from non-respiratory diseases, particularly cardiovascular ailments. Blood inflammatory factors are closely associated with chronic diseases and are commonly used in prognostic models.

Neutrophils serve as key mediators in the innate immune signaling in the lungs, capable of inducing inflammatory lung injury in both neonates and adults (4, 5). Simultaneously, neutrophils may become excessively activated in the airways of COPD patients, releasing inflammatory mediators such as interleukin-8, attracting more neutrophils to the affected areas, and initiating oxidative stress through the release of oxygen free radicals (6). Thus, neutrophils are considered crucial cell types in the pathogenesis of COPD. Lymphopenia is a marker of stress that can lead to alveolar destruction in COPD patients, with CD8+ cells producing pro-inflammatory cytokines including IL-2, interferon-gamma, and TNF-alpha, which are increased in COPD patients and recruit other inflammatory cells. Additionally, CD8+ cells can release perforins and granzyme B, causing cytolysis and apoptosis of alveolar epithelial cells, thereby promoting emphysema development. These inflammatory markers are easily derived from routine hematologic parameters (7–10). The neutrophil-to-lymphocyte ratio (NLR) indicates the balance between innate and adaptive immune responses, serving as an excellent indicator of inflammation and stress interaction. The opposite changes in neutrophil and lymphocyte counts represent a multifactorial dynamic process, depending on fine-tuning and regulation of various immune, neuroendocrine, humoral, and biological processes, such as margination/demargination, mobilization/redistribution, acceleration/retardation, and immune modulation (11). Therefore, NLR has garnered widespread attention in biomedical research both domestically and internationally and demonstrates good performance in predicting adverse outcomes in various disease states in clinical practice.

Currently, the use of NLR to predict all-cause mortality and cause-specific mortality in COPD has not been well established. Thus, this study includes representative data of COPD patients from the NHANES database from January 1999 to December 2018 and prospectively analyzes the association between NLR and the risk of all-cause and cause-specific mortality, aiming to provide reference for the clinical prevention and treatment of COPD.

## 2 Methods

### 2.1 Study design and population

The National Health and Nutrition Examination Survey (NHANES) is a large cross-sectional study aimed at assessing the nutritional status of non-institutionalized individuals in the United States. Data collection involves structured interviews conducted at home, physical examinations, and laboratory tests performed at mobile examination centers. The survey employs a multi-stage probability sampling design to select a representative sample of the U.S. population, assessing their health status every 2 years. Detailed methods can be obtained from the official website.^1^ Data analysis for all NHANES data from January 1999 to December 2018 utilized mobile examination center (MEC) examination data, considering stratification and clustering through the use of different weights for each year (wtmec4yr and wtmec2yr). This study protocol was approved by the Research Ethics Review Board of the National Center for Health Statistics (NCHS), and written informed consent was obtained from all NHANES participants, thus additional ethical approval was not required.

**Figure 1:**
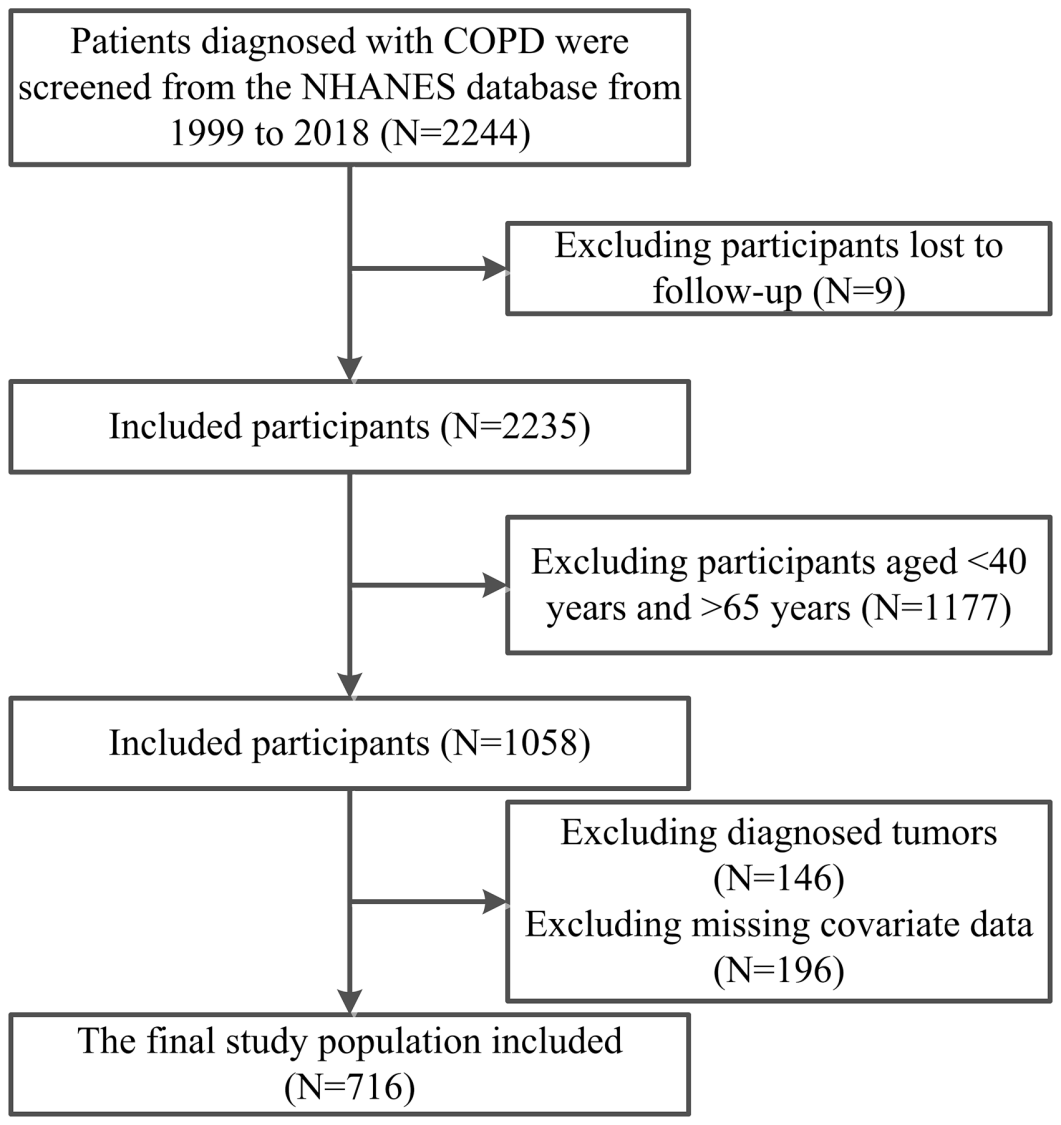
Flowchart of data inclusion and exclusion criteria.

This prospective cohort study utilized data from NHANES from January 1999 to December 2018. Exclusion criteria included age < 40 or > 65 years, patients diagnosed with tumors during cross-sectional surveys, patients without follow-up data, and patients with missing baseline information and covariates. The flowchart of participant inclusion and exclusion is depicted in Figure 1.

### 2.2 Definition of COPD and measurement of blood cell technologies

In order to accurately identify our target population, we employed three different diagnostic criteria, which could be implemented in the NHANES database. To enhance the accuracy of the population, constraints were added regarding smoking history and the use of COPD-related medications. Here, we describe each selected criterion (1): Forced expiratory volume in 1 s/forced vital capacity after bronchodilator use <0.7 (12); (2) Diagnosis of chronic obstructive pulmonary disease by a physician or other healthcare professional; (3) Age ≥ 40 years, taking COPD-related medications (including phosphodiesterase-4 inhibitors, mast cell stabilizers, combination bronchodilator therapy, etc.), and meeting any of the following conditions: smoking history of ≥100 pack-years, diagnosis of emphysema or chronic bronchitis, or still having chronic bronchitis. This identification strategy is widely used in the literature (13, 14).

Complete blood cell count is a routine blood test used to assess the overall health status of subjects and detect various diseases. The method for complete blood cell count is based on the Beckman Coulter method. The calculation method of NLR involves dividing the absolute neutrophil count by the absolute lymphocyte count from the same automated complete blood cell count sample.

### 2.3 The risk of mortality in the study population

The primary endpoint was the risk of all-cause mortality, determined using Mortality Linkage Files (MLF) recording death risks associated with any cause of death. These mortality risk data were obtained from the National Death Index (NDI) database.^2^ The follow-up time for each individual extended from the date of participation until the date of death or until December 31, 2019 (the latest update date of the NDI database). International Statistical Classification of Diseases, 10th Revision (ICD-10) codes were used to identify cases of cardiovascular mortality (I00-I09, I11, I13, and I20-I51).

### 2.4 Definition of primary variates

The covariates include (1) demographic characteristics: age, sex, race, marital status, education level, family income-poverty ratio (PIR), family insurance, smoking status, and alcohol consumption; (2) physical examination parameters: Body Mass Index (BMI); (3) medical conditions: hypertension, diabetes, cardiovascular disease. These variables are derived from the demographic and health questionnaires of the NHANES survey. Specifically, age is categorized as 40–54 years and 55–65 years; sex is categorized as male and female; race is categorized as white and other races; marital status is categorized as married/spouse-present and divorced/widowed; education level is classified as elementary school or below, high school or vocational school, and college or above; PIR is defined as the ratio of family income to the poverty line, categorized as low income (<1.3%), medium income (1.3% ≤ PIR ≤ 3.5%), and high income (>3.5%) (15, 16); insurance status is based on whether the patient has purchased family insurance according to the survey questionnaire; smoking status is categorized as never smoked (defined as smoking fewer than 100 cigarettes), former smoker (smoked more than 100 cigarettes but currently not smoking), and current smoker (smoked more than 100 cigarettes, currently smoking a few days or every day) (17); alcohol consumption status is categorized as never drank (defined as consuming fewer than 12 drinks in a lifetime), and drank (defined as consuming 12 or more drinks in a lifetime) (18); BMI is calculated as weight (kg) divided by height (m) squared, categorized as normal (<25 kg/m2), overweight (25 ≤ BMI < 30 kg/m2), and obese (≥30 kg/m2) (19); diabetes is defined as fasting blood glucose ≥7.0 mmol/L, self-reported diabetes diagnosis, or use of insulin or antidiabetic medications by the study participants (20); hypertension is defined as blood pressure measurement ≥140/90 mmHg, self-reported hypertension diagnosis, or use of antihypertensive medications by the study participants (20); CVD includes patients with coronary heart disease, congestive heart failure, heart attack, stroke, and angina pectoris (21).

### 2.5 Statistical analysis

According to NHANES analysis and reporting guidelines, complex sampling design and sampling weights (wtmec4yr and wtmec2yr) were considered during the analysis period. All variables have been converted from continuous variables to categorical variables, described using percentages, and assessed for intergroup differences using the chi-square test. The optimal NLR cutoff point associated with survival outcomes was obtained using the maximum selected rank statistics method based on the “maxstat” package (22, 23).^3^ Participants were then divided into high NLR and low NLR groups. Weighted Cox regression analysis was conducted to assess the correlation between NLR in COPD patients and the risk of all-cause and cardiovascular mortality. Three models were constructed to adjust for potential confounders. Model 1 adjusted for gender, marital status, race, age, and education level; Model 2 additionally adjusted for smoking, insurance, alcohol consumption, and PIR based on Model 1; Model 3 further adjusted for diabetes and cardiovascular disease based on Model 2.The probability of survival outcomes was calculated using the Kaplan–Meier method, and comparisons were made using the log-rank test. Subgroup analysis of NLR and COPD patient mortality risk was conducted among groups stratified by gender, age, marital status, race, smoking, BMI, and education level, analyzing their correlations. Restricted cubic spline (RCS) models were constructed to visualize the relationship between continuous NLR and outcomes. R statistical software, version 4.3.2,^4^ was used for analysis. Two-tailed *p* < 0.05 indicated statistical significance.

## 3 Results

### 3.1 The characteristics of the study population

**Figure 2:**
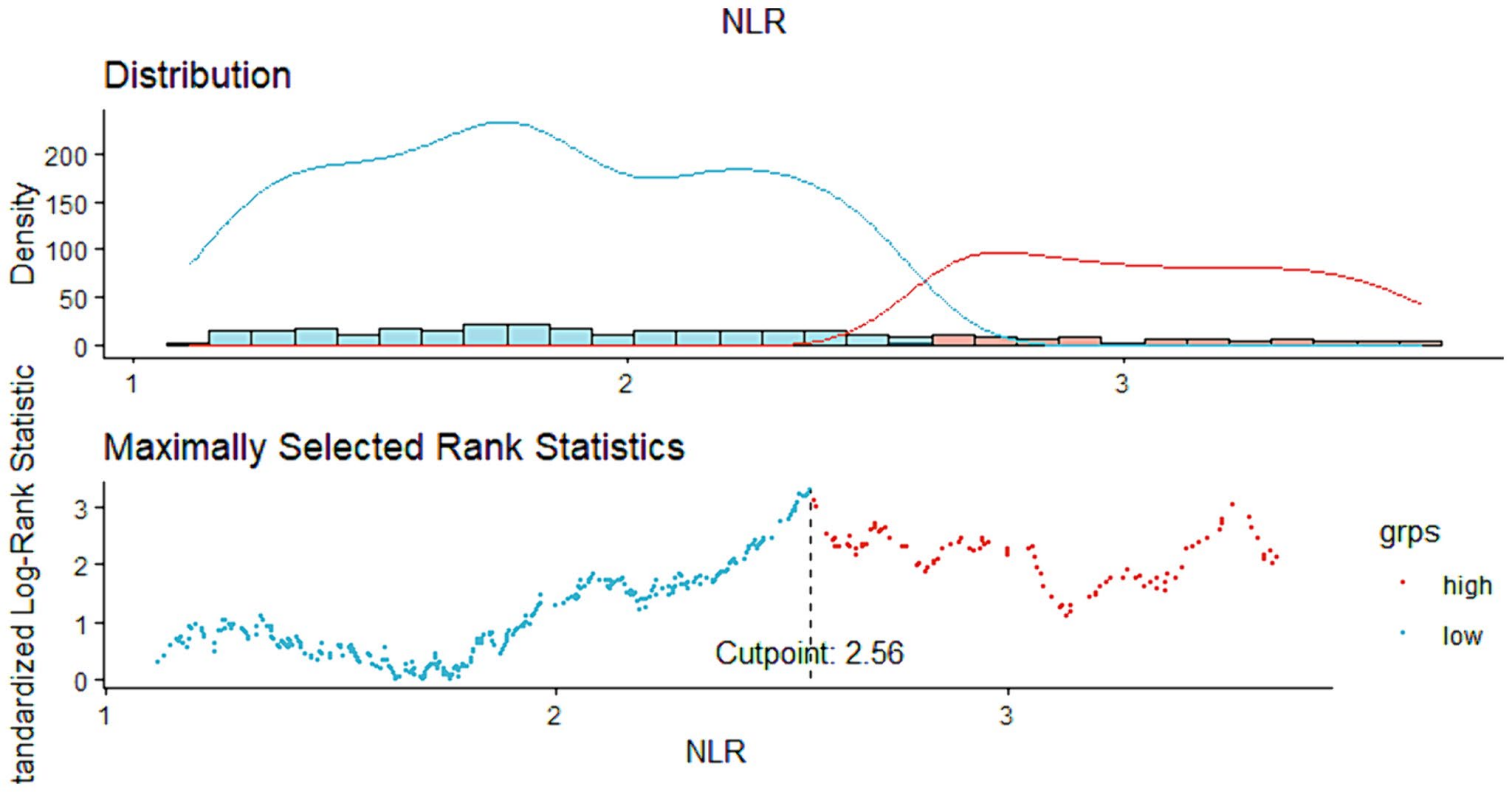
Cut-off points calculated using the ‘maxstat’ package, computed using standardized Log-Rank statistics.

The study ultimately included 716 COPD patients, and the optimal NLR cutoff value of 2.56 was determined based on the maximum selected rank statistics method, which showed the most significant correlation with survival duration. Participants were divided into high NLR (NLR ≥ 2.56, *n* = 208) and low NLR (NLR < 2.56, *n* = 508) groups (Figure 2).

Age, BMI, and PIR were continuous variables and were converted into categorical variables. There were no statistically significant differences between the high and low NLR groups in terms of age, sex, race, marital status, education level, family income-poverty ratio, health insurance, smoking status, alcohol consumption, BMI, hypertension, diabetes, and cardiovascular disease (all *p* > 0.05). These results indicate that NLR levels are not associated with age, sex, race, marital status, education level, family income-poverty ratio, health insurance, smoking status, alcohol consumption, BMI, hypertension, diabetes, or cardiovascular disease-related variables. Additional characteristics of the participants are provided in Table 1.

**Table 1:** Basic characteristics of COPD patients based on NLR classification.

Characteristic	Overall	Lower NLR	Higher NLR	*p*- value
	*n* = 716(%)	*n* = 554(%)	*n* = 162(%)	
Age group				0.44
40–54 years	325(49.00)	232(50.27)	93(46.35)	
55–65 years	391(51.00)	276(49.73)	115(53.65)	
Sex				0.1
Female	348(50.59)	264(53.12)	84(45.30)	
Male	368(49.41)	244(46.88)	124(54.70)	
Race/ethnicity				0.22
White race	428(80.70)	285(79.19)	143(83.88)	
Other race	288(19.30)	223(20.81)	65(16.12)	
Marital status				0.28
Married/with spouse	421(68.40)	300(70.05)	121(64.95)	
Divorced/widowed/living alone	295(31.60)	208(29.95)	87(35.05)	
Educational attainment				0.73
Junior high school or below	221(22.96)	157(22.31)	64(24.34)	
High school or technical secondary school	181(26.36)	123(27.45)	58(24.08)	
College or above	314(50.68)	228(50.25)	86(51.58)	
Poverty income ratio group				0.96
<1.3	304(30.49)	213(30.23)	91(31.04)	
1.3–3.5	229(32.66)	165(33.14)	64(31.67)	
> 3.5	183(36.85)	130(36.64)	53(37.29)	
Health insurance				0.71
Insured	591(85.31)	421(84.89)	170(86.18)	
Uninsured	125(14.69)	87(15.11)	38(13.82)	
Smoking status				0.6
Non-smoker	116(17.75)	88(19.06)	28(14.99)	
Former smoker	249(37.42)	177(36.90)	72(38.52)	
Current smoker	351(44.83)	243(44.04)	108(46.49)	
Alcohol consumption status				0.11
Yes	667(94.51)	473(95.54)	194(92.34)	
No	49(5.49)	35(4.46)	14(7.66)	
BMI(kg/m^2^)				0.74
Normal(< 25)	196(27.65)	141(28.56)	55(25.75)	
Overweight (25–30)	214(29.92)	152(30.23)	62(29.25)	
Obese(≥ 30)	306(42.43)	215(41.21)	91(44.99)	
Hypertension				0.12
Yes	369(46.24)	252(43.57)	117(51.86)	
No	347(53.76)	256(56.43)	91(48.14)	
Diabetes				0.6
Yes	134(15.10)	91(14.11)	43(17.19)	
Boardline	22(2.40)	18(2.57)	4(2.04)	
No	560(82.50)	399(83.32)	161(80.77)	
CVD				0.8
Yes	166(20.59)	112(20.26)	54(21.29)	
No	550(79.41)	396(79.74)	154(78.71)	

Continuous variables have been converted to categorical variables, with categories represented as numbers (%). All estimates have been adjusted based on sample weights and the complex survey design, further adjusted by NHANES survey weights. COPD stands for Chronic Obstructive Pulmonary Disease; BMI stands for Body Mass Index; NLR stands for Neutrophil-to-Lymphocyte Ratio.

### 3.2 Association between NLR and mortality

During a median follow-up of 111.5 months, among the 716 COPD patients, there were 163 deaths for calculating the NLR and all-cause mortality risk, while for assessing the NLR and cardiovascular mortality risk, a total of 603 COPD patients were included, with 49 deaths during the follow-up period. In model 1, adjustments were made only for gender, marital status, race, age, and education level. The results showed a significant correlation between the high NLR group as a continuous variable and the risk of all-cause mortality (HR = 1.21, 95% CI 1.12–1.29, *p* < 0.001) as well as cardiovascular mortality risk (HR = 1.16, 95% CI 1.01–1.34, *p* = 0.03). After adjusting for multiple factors, with each unit increase in NLR, the risk of all-cause mortality increased by 23% (model 2) and 20% (model 3), while the risk of cardiovascular mortality increased by 24% (model 2) and 31% (model 3). When evaluating NLR as a categorical variable, adjustments were made for demographic factors in model 1, including gender, marital status, race, age, and education level. The results indicated that the risk of all-cause mortality in COPD patients with high NLR group was twice that of the low NLR group (HR = 2.01, 95% CI: 1.39–2.92, *p* < 0.001), and the risk of cardiovascular mortality was 2.6 times higher than that of the low NLR group (HR 2.65, 95% CI: 1.38–5.07, *p* < 0.05). In model 2, adjustments were made for smoking, insurance, alcohol consumption, and PIR on the basis of model 1, and the results showed that these associations remained statistically significant. In model 3, further adjustments were made for BMI, insurance, hypertension, alcohol consumption, diabetes, and cardiovascular disease, and the positive correlation between the high NLR group and higher mortality risk persisted. Categorically, the risk of all-cause mortality in the high NLR group was 2.07 times that of the low NLR group, and the risk of cardiovascular mortality was 3.03 times that of the low NLR group (Table 2).

**Table 2:** Association of NLR with mortality risk in adults with COPD.

Characteristic	Model1	*p*	Model2	*p*	Model3	*p*- value
	HR (95%CI)		HR (95%CI)		HR (95%CI)	
All-cause mortality						
NLR (continuous variable)	1.21 (1.12–1.29)	<0.001	1.23 (1.16–1.31)	<0.001	1.20 (1.12–1.29)	<0.001
Higher NLR*	2.01 (1.39–2.92)	<0.001	1.98 (1.38–2.86)	<0.001	2.07 (1.46–2.94)	<0.001
Cardiovascular mortality						
NLR (continuous variable)	1.16 (1.01–1.34)	0.03	1.24 (1.05–1.47)	0.01	1.31 (1.11–1.55)	0.001
Higher NLR*	2.65 (1.38–5.07)	0.003	2.40 (1.24–4.65)	0.01	3.03 (1.63–5.65)	<0.001

In Model 1, confounders were adjusted for gender, marital status, race, age, and education level only; Model 2 was adjusted based on gender, marital status, race, age, education level, smoking, insurance, alcohol consumption, and PIR; Model 3 adjusted for gender, race, marital status, age, education level, smoking, PIR, BMI, insurance, hypertension, alcohol consumption, diabetes, and CVD. *Risk of mortality was analyzed compared to lower NLR group.

**Figure 3:**
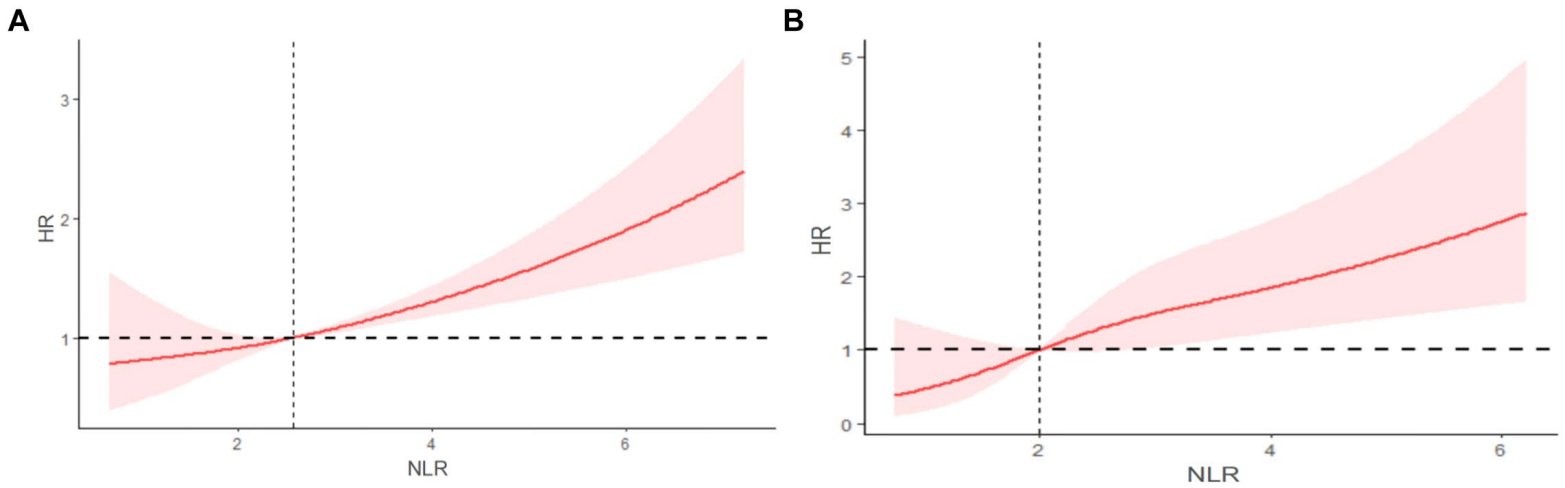
Restricted cubic spline (RCS) showing the relationship between NLR and all-cause mortality **(A)** and cardiovascular mortality **(B)** in COPD patients.

Kaplan–Meier survival curves showed that the risk of all-cause mortality (*p* = 0.0013) and cardiovascular mortality (*p* = 0.019) was higher in the high NLR group compared to the low NLR group, indicating lower survival rates in the high NLR group (Figure 3).

### 3.3 Stratified analyses

When conducting subgroup analysis based on age, gender, marital status, race, BMI, and education level, the high NLR group exhibited an increased risk of all-cause mortality and cardiovascular mortality compared to the low NLR group (HR > 1). Regarding the risk of all-cause mortality, the HR was less than 1.0 for the subgroup categorized as other races, which may be attributed to the relatively lower proportion of individuals in this racial category. When *p* > 0.05, it indicates no significant difference between the two groups. Several patient subgroups did not reach statistical significance, including the subgroup of other races in the race classification (*p* = 0.33), overweight individuals (*p* = 0.73), and individuals with a college education or above (*p* = 0.12). There were no significant interactions observed for gender (*p* = 0.42), age (*p* = 0.58), marital status (*p* = 0.71), education level (*p* = 0.65), and BMI (*p* = 0.17), while a significant interaction existed among races (*p* = 0.004). Regarding the risk of cardiovascular mortality, the HR was less than 1.0 for the subgroup categorized as other races, possibly due to the lower proportion of individuals in this racial category. Several patient subgroups did not reach statistical significance, including the subgroup of divorced/widowed/living alone in the marital status category (*p* = 0.46), other races in the race classification (*p* = 0.13), overweight individuals (*p* = 0.9), and individuals with a college education or above (*p* = 0.26). There were no significant interactions observed for gender (*p* = 0.36), age (*p* = 0.89), marital status (*p* = 0.08), education level (*p* = 0.6), and BMI (*p* = 0.05), while a significant interaction existed among races (*p* < 0.001; Table 3).

**Table 3:** Subgroup analyses of NLR and mortality risk in COPD.

Variable	All-cause mortality higher NLR (≥2.56)	*p*- value	*p*- for interaction	Cardiovascular mortality higher NLR (≥2.56)	*p*- value	*p*- for interaction
	HR (95%CI)			HR (95%CI)		
Sex			0.42			0.36
Male	1.75 (1.09–2.82)	0.02		2.26(1.11–4.59)	0.02	
Female	2.46 (1.30–4.66)	0.01		4.45(1.52–13.02)	0.01	
Age group			0.58			0.89
40–54 years	2.22 (1.09–4.50)	0.03		2.90(0.99–8.46)	0.05	
55–65 years	1.78 (1.11–2.85)	0.02		2.63(1.25–5.53)	0.01	
Marital status			0.71			0.08
Married/with spouse	2.19 (1.34–3.59)	0.002		3.80(1.80–8.02)	<0.001	
Divorced/widowed/living alone	1.88 (1.07–3.31)	0.03		1.37(0.59–3.17)	0.46	
Race/ethnicity			0.004			< 0.001
White race	2.53 (1.60–4.01)	<0.001		3.96(1.89–8.28)	<0.001	
Other race	0.69 (0.33–1.46)	0.33		0.27(0.05–1.49)	0.13	
BMI(kg/m^2^)			0.17			0.05
Normal(< 25)	2.76(1.50–5.09)	0.001		13.30(2.93–60.34)	<0.001	
Overweight (25–30)	1.13(0.56–2.31)	0.73		1.09(0.32–3.68)	0.9	
Obese(≥ 30)	2.47(1.38–4.42)	0.002		2.57(1.13–5.84)	0.02	
Educational attainment			0.65			0.6
Junior high school or below	2.13(1.17–3.87)	0.01		3.88(1.69–8.90)	0.001	
High school or technical secondary school	2.57(1.28–5.15)	0.01		3.41(1.04–11.22)	0.04	
College or above	1.69(0.88–3.27)	0.12		2.07(0.59–7.28)	0.26	

HRs were adjusted for sex, age group, race/ethnicity, BMI, and educational attainment. When analyzing sex, adjustments were made for the other variables, and so on for subsequent analyses.

These results indicate that the relationship between higher NLR and increased mortality risk persists in subgroups with potential confounding factors, and the racial differences associated with the correlation between NLR and the risk of all-cause mortality and cardiovascular mortality warrant further investigation.

### 3.4 Linear relationship of NPR and mortality

**Figure 4:**
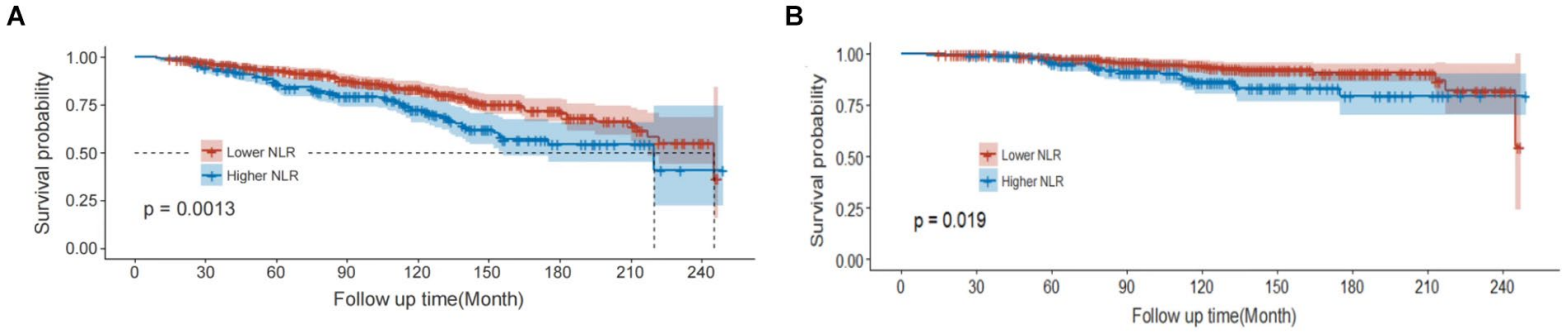
Kaplan–Meier curves of all-cause mortality **(A)** and cardiovascular mortality **(B)** among participates with COPD.

Figure 4A depicts the RCS curve illustrating the association between NLR and the risk of all-cause mortality. This curve, adjusted for confounding factors such as gender, race, marital status, age, education level, smoking, PIR, BMI, insurance, hypertension, alcohol consumption, diabetes, and CVD, describes a linear relationship between increasing NLR and the risk of all-cause mortality, with no statistical evidence of non-linearity (P non-linear = 0.7598). Figure 4B shows the RCS curve illustrating the association between NLR and the risk of cardiovascular mortality. Similarly, this curve, adjusted for confounding factors, describes a linear relationship between increasing NLR and the risk of cardiovascular mortality, with no statistical evidence of non-linearity (P non-linear = 0.531).

## 4 Discussion

This study, based on survey data from 716 COPD patients from the NHANES cycles (1999–2018), found a positive correlation between NLR and the risk of all-cause mortality and cardiovascular mortality in COPD patients across multiple models. The study identified the optimal NLR cutoff value as 2.56, making it an independent risk factor for poor survival in COPD patients. Therefore, incorporating NLR into routine clinical laboratory tests for COPD patients can effectively predict the risk of all-cause mortality and cardiovascular disease mortality.

NLR, as an easily accessible and widely used biomarker, has been employed in many studies to assess inflammatory status, predict the progression of chronic diseases, and evaluate the overall health status of patient. High NLR is often associated with the severity and poor prognosis of inflammatory diseases and malignanciess (24). In the context of malignant tumors, NLR has been shown to be an independent factor affecting the prognosis of various solid tumors, including lung cancer, colorectal cancer, pancreatic cancer, breast cancer, ovarian cancer, and gastric cancer (25–28). Additionally, NLR is closely related to the severity, hospitalization rate, malnutrition, recurrence, and mortality risk of chronic diseases such as cardiovascular and renal diseases (29, 30).

Templeton et al. demonstrated in their meta-analysis that an NLR above 4 could independently predict shorter overall survival in many cancers (31). Furthermore, Alice Drăgoescu et al. found in a prospective single-center study of septic patients admitted to the Intensive Care Unit (ICU) that NLR was elevated in all septic patients and significantly increased in septic shock patients (32). NLR showed good sensitivity (47%) and specificity (78%), with an area under the curve (AUC) of 0.631 (*p* < 0.05), suggesting its potential value in assessing the severity of sepsis, particularly when its value exceeds 10. In a large cohort study of 12,862 COVID-19 patients, NLR has been proposed as a guide to predict the efficacy of corticosteroid therapy. Specifically, patients with an initial NLR value >6.11 had a reduced risk of severe illness and 60-day mortality after receiving corticosteroid treatment. Conversely, patients with an initial NLR value ≤6.11 did not benefit significantly from corticosteroid therapy (33).

There is existing research suggesting that inflammation plays a crucial role in the onset and progression of COPD (34). Based on the above findings, it can be inferred that by monitoring NLR, researchers can assess the inflammatory status within patients and estimate their survival condition accordingly. This study, by investigating the association between NLR and the risk of all-cause mortality and cardiovascular mortality in COPD patients, has identified the clinical value of NLR in assessing these risks.

The study found that NLR can effectively predict the prognosis of COPD patients and has an independent impact on all-cause mortality and cardiovascular mortality. After determining the optimal NLR cutoff value as 2.56 using the maximum selection rank statistics, the study divided patients into high and low NLR groups. Across three models adjusting for potential confounders, the high NLR group exhibited increased mortality risk compared to the low NLR group. Baraldo et al. previously found that neutrophil infiltration was more correlated with airway smooth muscle inflammation in COPD patients than in healthy subjects (30). Additionally, the number of inflammatory cells of the main pathogenic type in COPD increased with its severity, with neutrophil increase seen in stage III and IV COPD patients (35). A significant feature of COPD is the enhanced or abnormal inflammatory immune response of the lungs to inhaled particles and gasses, with increased neutrophils, activated macrophages, and T lymphocytes (Tc1 and Th1 cells) among its main characteristics (36). Jasper et al. demonstrated that neutrophils are important effector cells in chronic obstructive pulmonary disease (37). Elevated NLR is positively correlated with the risk of death in hospitalized patients and may serve as an effective predictor of AECOPD (38).

In the search for predictive biomarkers for COPD, studies have examined the predictive role of immune cell activity and inflammation. Gao et al. showed in a longitudinal cohort study of 1,549 American veterans that NLR was associated with declining lung function, representing COPD risk. NLR was found to be a clinically relevant biomarker associated with lung function impairment and high COPD risk (OR = 1.07, 95% CI: 1.07–1.51) (39). Higher NLR levels were associated with frequent exacerbations and adverse outcomes in AECOPD patients, although the study did not identify a clinically significant cutoff value for NLR. In a retrospective study by Yao et al. involving 303 AECOPD patients, NLR and PLR levels were significantly higher in non-survivors compared to survivors of AECOPD. The study found a critical value of NLR to be 6.24, with the highest sensitivity (81.08%) and specificity (69.17%) for predicting in-hospital mortality risk when NLR was at the critical value (AUC = 0.803). When the critical value was 182.68, the sensitivity of PLR was 64.86%, the specificity was 58.27%, and the AUC was 0.639. The combined use of NLR, PLR, and CRP can improve prognosis sensitivity (40). However, the lack of adjustment for confounding factors, single-center, small sample size studies, and bias in patient selection and information may reduce the association with AECOPD outcomes, and the study was limited to hospitalized patients, lacking inclusion of the community population.

Building upon the existing research and addressing its limitations, this prospective cohort study of 716 community-based COPD patients found a significant positive correlation between elevated NLR and the risk of all-cause mortality and cardiovascular mortality. The potential mechanism may involve increased neutrophils causing systemic and respiratory system chronic inflammation (28), while decreased lymphocytes lead to decreased immune defense and resistance to disease (41).

Furthermore, in this study, after adjusting for confounding factors, the overall survival outcomes were worse in the high NLR group (≥2.56), with the optimal threshold defined by the maximum selection rank statistics. Kaplan–Meier survival curves were drawn based on the study data, indicating that patients in the high NLR group had higher risks of all-cause mortality and cardiovascular mortality than those in the low NLR group, with statistical significance. The relationship between higher NLR and higher mortality risk remained in subgroups of potential confounding factors. Therefore, we believe that NLR can be used to predict the risk of all-cause mortality and cardiovascular mortality in COPD patients, serving as a repeatable, easily accessible, low-cost, and reliable laboratory indicator. The strengths of this study lie in its large sample size of COPD patients with long-term follow-up and its prospective design, providing reliable data support for the study conclusions. In addition, all COPD patients came from the NHANES survey, preventing selection bias.

However, there are several limitations to this study. First, although adjustments were made for numerous potential confounding factors, there may still be unmeasured and unaccounted confounders, such as COPD complications and medication use, which may affect the observed results. Secondly, NLR was measured at a single time point, which may not reflect changes over time or in response to interventions. Therefore, the causal inference of this study is limited, and continuous NLR measurements can more accurately reflect inflammatory status. Finally, the study population analyzed included only adults aged ≥40 and ≤ 65 years with COPD in the United States, which may limit the generalizability of the conclusions to other regions and ethnicities and necessitate further research in different populations to validate our findings.

To establish the causal relationship between NLR and the risk of all-cause mortality and cardiovascular mortality in COPD patients, future studies should be conducted using multi-center, large-sample domestic data to mitigate selection and confounding biases and obtain complete follow-up data, thereby conducting randomized controlled trials to explore the effectiveness of NLR in predicting adverse outcomes in COPD patients. Nonetheless, the advantages of this nationally representative large-sample, long-term follow-up study and robust adjustment for confounding factors enhance the reliability of the results.

## 5 Conclusion

NLR is a potential laboratory indicator with significant implications for predicting all-cause mortality risk and cardiovascular mortality risk in COPD patients. Therefore, incorporating NLR into routine clinical tests to predict these risks in COPD patients can help identify high-risk individuals early for timely intervention to improve COPD prognosis.

## Data Availability

The original contributions presented in the study are included in the article/supplementary material, further inquiries can be directed to the corresponding author/s.

## Footnotes

^1^http://www.cdc.gov/nchs/nhanes.htm, accessed on October 1, 2023.

^2^ https://www.cdc.gov/nchs/data-linkage/mortality-public.htm

^3^ https://CRAN.R-project.org/package=maxstat

^4^ http://www.r-project.org
